# The Plastid Genome of Mycoheterotrophic Monocot *Petrosavia stellaris* Exhibits Both Gene Losses and Multiple Rearrangements

**DOI:** 10.1093/gbe/evu001

**Published:** 2014-01-06

**Authors:** Maria D. Logacheva, Mikhail I. Schelkunov, Maxim S. Nuraliev, Tagir H. Samigullin, Aleksey A. Penin

**Affiliations:** ^1^M.V. Lomonosov Moscow State University, Moscow, Russia; ^2^A.A. Kharkevich Institute for Information Transmission Problems, Russian Academy of Sciences, Moscow, Russia; ^3^V.A. Engelhardt Institute of Molecular Biology, Russian Academy of Sciences, Moscow, Russia; ^4^Joint Russian–Vietnamese Tropical Scientific and Technological Center, Cau Giay, Hanoi, Vietnam

**Keywords:** plastid genome, mycoheterotrophy, Petrosaviales, gene loss, genome rearrangements

## Abstract

Plastid genomes of nonphotosynthetic plants represent a perfect model for studying evolution under relaxed selection pressure. However, the information on their sequences is still limited. We sequenced and assembled plastid genome of *Petrosavia stellaris*, a rare mycoheterotrophic monocot plant. After orchids, *Petrosavia* represents only the second family of nonphotosynthetic monocots to have its plastid genome examined. Several unusual features were found: retention of the ATP synthase genes and *rbcL* gene; extensive gene order rearrangement despite a relative lack of repeat sequences; an unusually short inverted repeat region that excludes most of the rDNA operon; and a lack of evidence for accelerated sequence evolution. Plastome of photosynthetic relative of *P. stellaris*, *Japonolirion osense*, has standard gene order and does not have the predisposition to inversions. Thus, the rearrangements in the *P. stellaris* plastome are the most likely associated with transition to heterotrophic way of life.

Nonphotosynthetic plants represent a unique model for studying the evolution of plastid genome under relaxed selection. Typical plastome of photosynthetic plant contains ∼110 genes, and at least one-third of them encode proteins directly involved in photosynthesis. Apparently, a wide diversity of structures of plastid genomes, differing in gene content and order, should be observed in nonphotosynthetic plants—as it occurs in systems with experimentally induced heterotrophy (e.g., Cahoon et al. 2003). It seems, however, that only a limited number of ways of plastome modification was realized in the evolutionary history of higher plants. This may reflect some functional constraints or be just a consequence of insufficient sampling. But now only few complete plastome sequences, that of liverwort *Aneura mirabilis* (Wickett et al. 2008), parasitic dicots *Epifagus virginiana* and *Cistanche deserticola* (Wolfe et al. 1992; Li et al. 2013), and three mycoheterotrophic orchids (Delannoy et al. 2011; Logacheva et al. 2011; Barrett and Davis 2012), are available so this question is hard to address. *Corallorhiza striata* (Barrett and Davis 2012) demonstrates the least degree of reduction as its plastome is only 6% smaller compared with its photosynthetic relatives and *Rhizanthella* shows the highest degree—it has a plastome that is reduced by more than 50% compared with its relatives (Delannoy et al. 2011). In terms of gene content, in plastomes of nonphotosynthetic plants, chlororespiratory genes and most photosynthesis-related genes are lost or pseudogenized. The degree of reduction of other classes of genes is different; the most conserved are those that encode products involved in translation (ribosomal RNAs, ribosomal proteins, and transfer RNAs). Despite the drastic differences in length and gene content, these genomes are mainly colinear to that of photosynthetic plants, with the exception of minor shifts in the inverted repeat (IR)-single copy (SC) region boundaries. The information about the sequence of plastid genomes in nonphotosynthetic plants is poor mostly due to technical limitations. Tools that facilitate the analysis of plastid genome sequences were developed, including universal primer sets for amplification and sequencing (Heinze 2007; Dong et al. 2013) and computational resources (Wyman et al. 2004; Cheng et al. 2013). However, most of them are applicable mainly to the plastids that have “standard” gene content and order and not to the highly reduced and/or rearranged genomes that are expected for nonphotosynthetic plants. Also, many nonphotosynthetic plants are very small and represent rare species; this complicates the extraction of plastid DNA in a sufficient quantity. In last years, new DNA sequencing techniques made great progress, allowing overcoming these difficulties. The studies encouraging the researchers to use whole genome sequencing data to characterize organelle genomes are emerging (Smith 2012; Straub et al. 2012), and this approach can be applied to nonphotosynthetic plants as well.

In this study, we report the characterization of the complete plastome sequence of a mycoheterotrophic plant *Petrosavia stellaris* and partial sequence of its photosynthetic relative, *Japonolirion osense*, based on whole genome sequencing data using Illumina technology. The genus *Petrosavia* is very unusual in many respects, and it was treated as a sole representative of the family Petrosaviaceae (Cronquist 1981), but molecular studies revealed the affinities of *Petrosavia* and a monotypic endemic Japanese genus *Japonolirion* and they were united within one family (Cameron et al. 2003). Further insights from morphology supported this (Remizowa et al. 2006). Petrosaviaceae (including *Japonolirion*) have an isolated position within the monocots and are the sister group of all monocots except Alismatales and Acorales (Chase et al. 2006; Davis et al. 2006) and are treated within a monotypic order Petrosaviales (Angiosperm Phylogeny Group 2009). The loss of photosynthetic activity arose many times in evolution of monocots: besides Petrosaviales it is known in Pandanales, Asparagales, Liliales, and Dioscoreales (Merckx and Freudenstein 2010). Complete plastome sequences are available only for Orchidaceae; the examples from other monocot families are useful to understand the pattern of evolutionary transformations of plastid genomes under the loss of photosynthetic activity. Also, the information on plastid genome sequence from *Petrosavia* will improve the reconstruction of angiosperm phylogeny as Petrosaviales is one of the few angiosperm orders for which complete plastome sequence is not available.

A total number of 40,001,803 100-bp paired reads were generated for *Petrosavia* and 8,418,085 reads for *Japonolirion* total genomic DNA libraries (data are available in NCBI under Bioproject accession numbers PRJNA196233 and PRJNA196234 correspondingly). For *Petrosavia*, assembly by Velvet resulted in a single scaffold with high similarity to plastid genome. PCR joining and sequencing of the amplicons allowed reconstructing the complete sequence. As the de novo assembly algorithms are not able to distinguish between two copies of IR and we expected them to be assembled together, the position of IR region was deduced based on coverage (supplementary fig. S1, Supplementary Material online). Verification of the assembly was performed in three ways: 1) back-mapping of the reads on the assembled sequence used as reference, 2) comparison with sequences available in the GenBank, and 3) Sanger resequencing of several regions. All three methods confirmed consistent and accurate assembly. Approximately 321 thousands of reads were mapped as paired, with no zero coverage regions and an average coverage 346.9×. The sequences of *Petrosavia* plastid genes available from the GenBank (AF206806, AF209649, AY465613, AY465715, AY465690, AB088839, AB040156) aligned on our assembly at their total length and had 98–99% similarity for *P. stellaris* sequences and 96–99% for other *Petrosavia* species. The Sanger resequencing of selected regions (*petD*, *rps16-trnQ-UUG*, *ycf2*, *atpA*, *trnL-UAA-trnF-GAA*) using the same DNA sample as for Illumina sequencing yielded 100% similar sequences.

Complete sequence of *P. stellaris* plastid genome represents a circular molecule 103,835 bp in length, with the IR 10,750 bp, large single copy (LSC) 62,725 bp, and small single copy (SSC) 19,610 bp (GenBank accession number KF482381). GC content: total 37.47, LSC 36.39%, SSC 40.47%, IR 37.9%. In terms of gene content, it encodes a reduced gene set represented for the most part by genes responsible for protein synthesis—all ribosomal protein as well as ribosomal and transfer RNA genes (except for the *trnT-GGU* which is present as pseudogene) are intact. Also, two giant plastid genes—*ycf1* that encodes a component of plastid translocon complex (Kikuchi et al. 2013) and *ycf2* (unknown function) and genes involved in plastid metabolism (*accD* and *clpP*)—are conserved. The genes related to photosynthesis are either lost or pseudogenized, with exception of *rbcL*, *psbZ*, *petG*, and genes encoding subunits of ATP synthase (fig. 1). The genes that are retained have high similarity with those of photosynthetic monocots (table 1). Positions of introns in splitted genes are conserved. Sequencing of cDNA of two intron-containing genes—*clpP* and *rps12* (accession numbers KF482379 and KF482380, respectively)—confirmed the presence of spliced transcripts. The *rpl2* gene has an atypical start codon ACG—a feature shared between all monocots, both photosynthetic and nonphotosynthetic. cDNA sequencing showed the presence of C/T polymorphism at the second position of *rpl2* start codon (supplementary fig. S2, Supplementary Material online), indicating the presence of RNA editing.

**Fig. 1.—:**
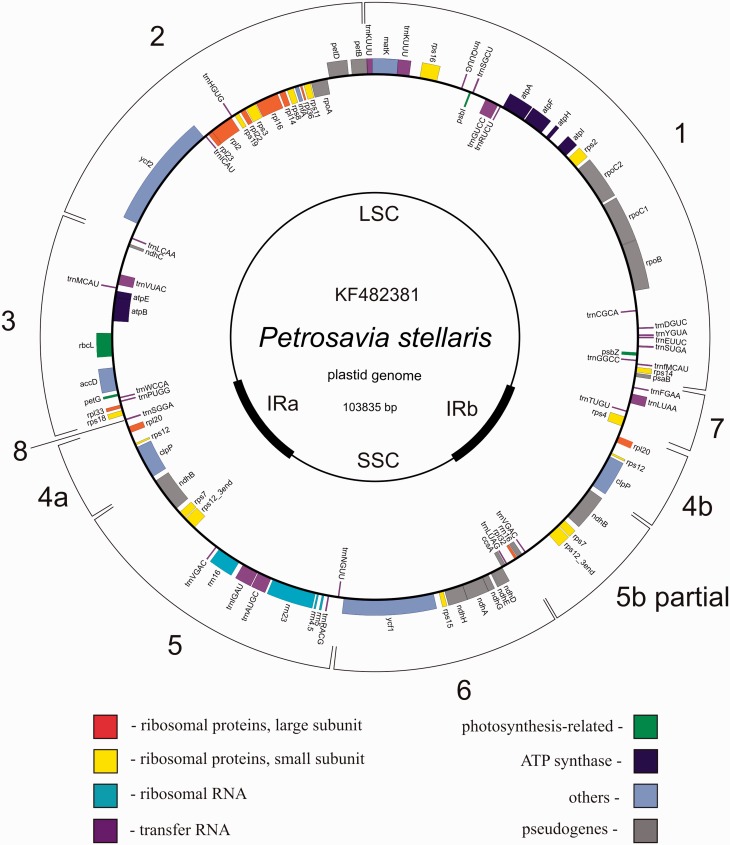
Circular map of the plastid genome of *Petrosavia stellaris*. Genes shown inside the circle are transcribed clockwise, those outside the circle are transcribed counterclockwise. Numbered curves outside of the map outline blocks colinear to non-rearranged plastid genomes.

**Table 1:** Conservation of Genes in *Petrosavia stellaris* Plastome

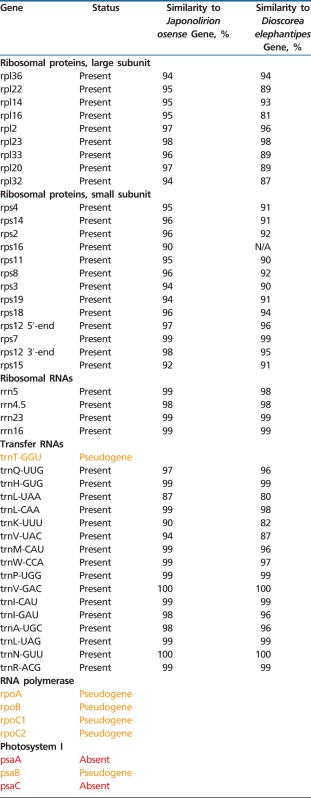
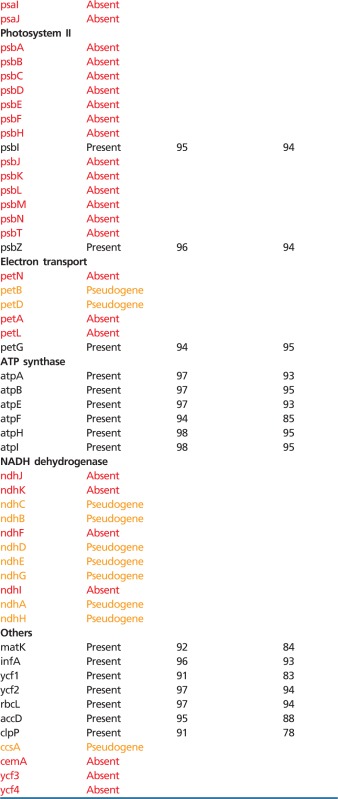

Note.—Similarity is calculated based on pairwise alignments of complete gene sequences (for splitted genes including introns).

The most unusual trait is the gene order, including the position of IR. The LSC-IR junction is located between the genes *rps4*–*rpl20* from one side and *rpl20*–*rps18* from the other. SSC-IR junction lies within *rrn16* gene, and all other *rrn* genes are located in the single copy region. *Petrosavia* plastome is highly rearranged relatively to the plastomes of other monocots. There are seven major syntenic blocks: from *trnK* to *psaB* (block 1), from *petB* to *trnL-CAA* (block 2), *ndhC-rps18* (block 3), *rpl20-clpP* (block 4), *ndhB-trnN-GUU* (block 5), *ycf1-rpl32* (block 6, encompasses the SSC), *trnF-GAA-rps4* (block 7), and a block 8 represented by single gene, *trnS-GGA* (fig. 1).

For *Japonolirion*, assembly resulted in seven contigs longer than 1 kb with total length 128,505 bp (supplementary table S1, Supplementary Material online). The quality of DNA, isolated from 10-year-old ethanol-fixed material, was insufficient for using PCR to join all the contigs. Thus, the comparative analysis was done using four longest contigs (52,466, 26,533, 23,498, and 18,560 bp). These contigs contain more than 90% of genes typical for plastid genome of a photosynthetic plant. Based on coverage and gene content, we attribute 52,466, 23,498, and 18,560 bp contigs to single copy regions and the 26,533 bp contig to IR region. Comparison with other monocots shows that gene order in these contigs does not deviate from the typical. Thus, we assume that *Japonolirion* possesses a non-rearranged plastome. Based on this assumption, we propose that the following events mediated transition from ancestral, non-rearranged plastid genome to that observed in *P*. *stellaris*: 1) large inversion in the LSC affecting the *trnK-rps4* region (blocks 1-8-7), 2) contraction of the IR to *ndhB*–*rrn16* (block 5b partial), 3) translocation of *ndhC-clpP* (blocks 3-4a) into *trnL-CAA-ndhB* spacer (junction of blocks 2 and 5), 4) expansion of the IR to include *clpP-rpl20* (block 4), and 6) translocation of *trnS-GGA* (block 8) between blocks 3 and 4a.

As relationships of *Petrosavia* to other monocots were never studied using plastid genome-scale data, we employed the information from plastid genome for phylogenetic reconstruction. Trees inferred from nucleotide and aminoacid sequences are mostly congruent, with few exceptions confined to poorly supported nodes. *Petrosavia* is sister to *Japonolirion* (with 100% support), and *Petrosavia* + *Japonolirion* clade is sister to all monocots except for Alismatales and Acorales (fig. 2). This is consistent with the results of analysis of small (2–4) number of genes but high number of taxa (including *Petrosavia*) (Chase et al. 2006) and larger plastid data sets where Petrosaviales are represented by *Japonolirion* only (Barrett et al. 2013; Davis et al. 2013).

**Fig. 2.—:**
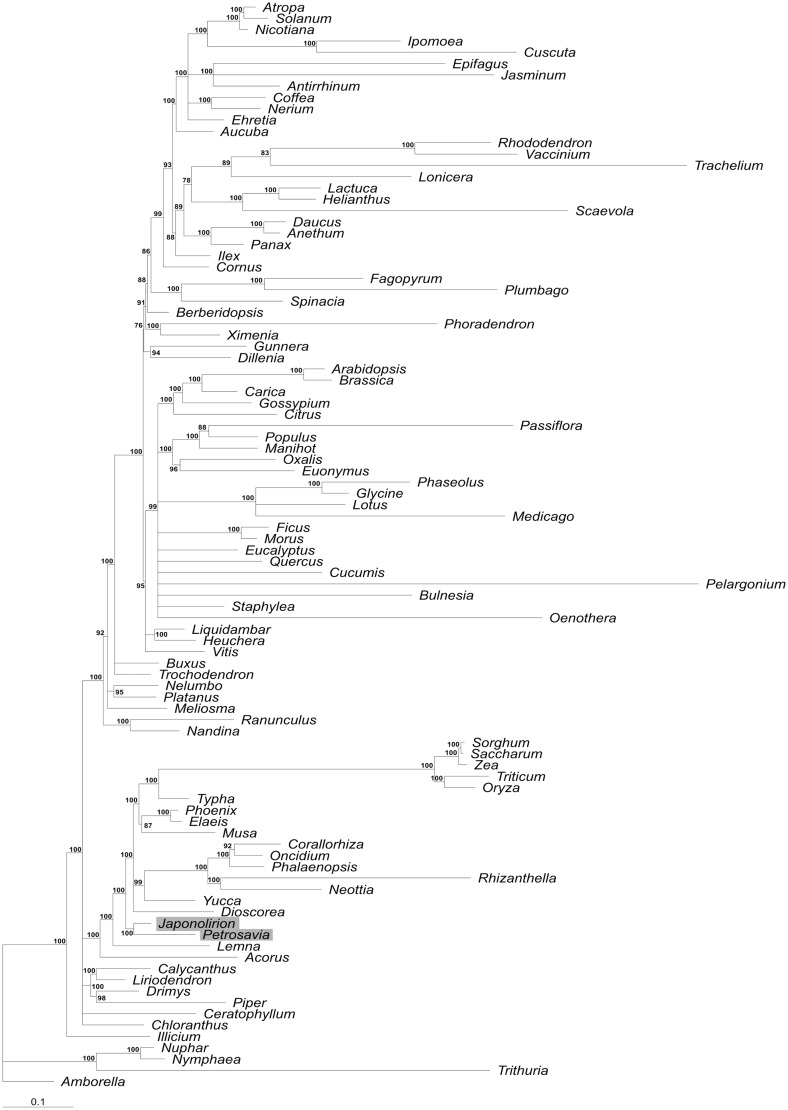
Phylogenetic tree inferred by RAxML using nucleotide sequences of 37 protein coding genes shared between 93 angiosperm plastid genomes. Branch length is proportional to number of nucleotide substitutions. Numbers above nodes indicate bootstrap values. Nodes with support less than 50% are collapsed.

Nonphotosynthetic plants often exhibit the increased rate of nucleotide substitutions in all their genomic compartments (Bromham et al. 2013). The same is characteristic for organelles of some photosynthetic species, especially those with rearranged plastomes (Guisinger et al. 2008; Sloan et al. 2012). We compared nucleotide substitution rates in *Petrosavia* and in other flowering plants. Analysis of relative nucleotide substitution rates shows no increased rate in *Petrosavia* (supplementary table S2, Supplementary Material online). Although the relative nucleotide substitution rate in *Petrosavia* is higher than that in many other monocots (including *Japonolirion*), it is considerably lower than in other nonphotosynthetic plants (e.g., in *Epifagus* it is 3.6 times higher, in *Neottia* and *Rhizantella* 2.4 and 5.5 times) or photosynthetic plants with rearranged plastid genomes (in *Pelargonium*, *Trachelium*, *Scaevola* 3.9–4.5 times higher).

Similar to other nonphotosynthetic plants, *Petrosavia* plastome has lost most of photosynthesis-related genes. Patterns of gene loss are generally consistent with the order proposed by Barrett and Davis (2012). They suggested, based on their observations on plastid genomes of mycoheterotrophic orchids, that the *ndh* genes are the most susceptible to loss and the *atp* are the least. *Petrosavia* seems to be at early stages of plastome degradation as the gene set is much more complete than that of *Epifagus*, *Neottia*, and *Rhizanthella* and is similar to that of *Corallorhiza*. The conservation of *rbcL* in *Petrosavia*, unexpected for a nonphotosynthetic plant, was also observed in several holoparasitic species (Delavault et al. 1995; Randle and Wolfe 2005). This may be explained by either recent loss of photosynthetic ability or by existence of alternative functions of *rbcL* gene product in these plants (Krause 2008). Another distinctive feature of *Petrosavia* plastome is the high number of rearrangements. The most plausible explanation is that these rearrangements occur as a result of relaxed selection caused by switch to heterotrophy. However, the plastomes of nonphotosynthetic plants characterized by date are colinear to that of their photosynthetic relatives, even in case of extreme reduction seen in *Rhizanthella* (Delannoy et al. 2011). On the other hand, there are several examples of extensive rearrangements of plastomes which all occur in plants with no signs of impairment of photosynthetic function—in Campanulaceae (Haberle et al. 2008), Geraniaceae (Guisinger et al. 2011), and Fabaceae (Cai et al. 2008). In most cases where rearrangements were found in photosynthetic plants’ plastomes, they were correlated with the highly increased number and length of repeats. The putative mechanism generating them is the intramolecular recombination between these repeats. Now about 200 complete plastid genome sequences are available for flowering plants, representing all major evolutionary lineages within this group. This allowed us to perform a global survey of the repeat content and its correlation with the conservation of gene order (supplementary table S3, Supplementary Material online). In basal angiosperms, magnoliids, and basal eudicots, plastid genomes have low number of repeats and show no or minor deviations from the typical gene order. There are, however, several reports of IR/SC boundary shifts and inversions in Ranunculaceae (e.g., Johansson and Jansen 1993), thus apparent uniformity of plastid genomes in basal eudicots might be result of undersampling. In rosids, most species also have low number of repeats and typical gene order, but there are notable exceptions found in the families Geraniaceae and Fabaceae where rearrangements are abundant (up to 16 colinear blocks in *Trifolium subterraneum*). As mentioned earlier, this trait is well documented and studied in details in both families and is found to be correlated with the high number of repeats and increased evolutionary rates (Chumley et al. 2006; Guisinger et al. 2008, 2011; Magee et al. 2010). All these three features are hypothesized to be caused by aberrant DNA repair (Guisinger et al. 2008). In asterids, highly rearranged plastomes are found in Campanulaceae (Cosner et al. 2004; Haberle et al. 2008) and Ericaceae (Fajardo et al. 2013). In both cases, high number of repeats is observed. In *Petrosavia*, repeat content is low so it is unlikely that its photosynthetic ancestor could have high repeat content. Also, no increase in substitution rate is found; this suggests that the mechanisms responsible for rearrangements are different in photosynthetic dicots and in *Petrosavia*. Thus, the characterization of *Petrosavia* plastome demonstrates that despite the increased knowledge on plastid genomes, an important modus of nonphotosynthetic plastomes’ evolution, related to genome rearrangements, remained overlooked.

Besides information on *Petrosavia* plastid genome structure, our study provides an example of de novo assembly of organellar genome from low-coverage nuclear genome sequence data for nonphotosynthetic plant. This approach can be used not only for plastid genome, but we were also able to assemble partial sequence of mitochondrial genome—the assembly produced 38 scaffolds with total length ∼840 Kb which have high similarity to plant mitochondrial genomes (will be presented elsewhere). The retrieval of the data on organelle (mainly chloroplast) genomes from short-read high throughput sequencing data is not novel (e.g., Wang and Messing 2011). For nonphotosynthetic plants, this approach was used only once, and it employed the information on the plastome structure in the related species for the alignment of contigs resulting from de novo assembly and further iterative gap closing (Barrett and Davis 2012). Any deviations from the typical gene order, including shift of IR regions and rearrangements, impede the application of this approach. We found that the de novo assembly generates long and accurate contigs of plastid genomes that can be joined into complete sequence using PCR without relying on the information about plastomes of related species. However, there is an important precondition for the successful assembly of the organellar genomes—the gap between the coverage of plastid genome and mitochondrial genome. Mitochondrial genomes harbor many sequences of plastid origin; the reverse situation is much rarer but also occurs (Iorizzo et al. 2012). This creates a threat of generation of incorrect contigs chimeric between plastid and mitochondrial genomes. In case if there is a great difference between read depth of plastid and mitochondrial genomes, it is possible to reveal such misassemblies by analyzing the contig coverage. Typically, the coverage of the plastid genome is much higher than for the mitochondrial one, because of its smaller size and higher copy number per cell (Straub et al. 2012). We observed the same situation in *Petrosavia*, where the coverage of plastid genome is about 350× and that of contigs derived from mitochondrial genome is about 40×. We expect that the same could be applied for other nonphotosynthetic plants.

## Materials and Methods

Plant material of *P. stellaris* was collected by M.S. Nuraliev during expedition of Russian-Vietnamese Tropical Centre in Vietnam in spring 2012 (voucher information: Russian-Vietnamese Tropical Centre, Petrosavia stellaris Becc., Dak Lak prov., Lak distr., Bong Krang municipality, Chu Yang Sin National Park, 14 km S from Krong Kmar village, in mixed forest, on the mountain ridge N 12° 22′ 45″ E 108° 21′ 25″ alt. 1,800 m, Nuraliev M.S. No. 486, April 6, 2012 det. D.D. Sokoloff). An additional sample was collected in the same location by M.S. Nuraliev, A.N. Kuznetsov, and S.P. Kuznetsova in 2013 and fixed in RNAlater (Ambion, USA) in order to preserve RNA. To characterize *J. osense* plastome, we used ethanol-fixed material (Japan, Gunma prefecture, Mount Shibutsu, collected by I.V. Tatarenko on July 4, 2003). Total genomic DNA from both samples was extracted from a single plant using CTAB method (Doyle and Doyle 1987) with two modifications: 1) we used pure chloroform instead of chloroform-isoamyl alcohol and 2) chloroform extraction was performed twice. To construct the libraries for whole genome sequencing, DNA was processed as described in the TruSeq DNA Sample Preparation Guide (Illumina). Libraries were quantified using fluorimetry with Qubit (Invitrogen, USA) and real-time PCR and diluted up to final concentration of 9 pM. Diluted libraries were clustered on a paired-end flowcell using cBot instrument and sequenced using HiSeq2000 sequencer with TruSeq SBS Kit v3-HS (Illumina, USA), read length 101 from each end. The assembly of *P. stellaris* plastome was performed by Velvet 1.2.03 (Zerbino and Birney 2008) using 5 million read pairs (10 million reads) with k-mer length 65 and expected k-mer coverage (exp_cov parameter) 150. Assembly of *J. osense* plastome was performed using CLC Genomics Workbench v. 5.5 with following parameters: word size = 22, bubble size = 50, mismatch cost = 2, insertion cost = 3, deletion cost = 3, minimal contig length = 1,000 bp. Based on assembly, primers were designed to join contigs (supplementary table S4, Supplementary Material online). PCR was run on MJ Mini thermal cycler under the following conditions: initial denaturation 90 s at 95 °C, then 32 cycles of denaturation 10 s at 95 °C, primer annealing 25 s at 56–60 °C depending on primer GC-content, and elongation 40–120 s at 72 °C. All reactions were performed using reagent from Encyclo PCR kit (Evrogen, Russia) following manufacturer’s instructions. To check the presence of spliced transcripts and RNA editing, we extracted RNA from RNAlater-fixed material using RNEasy Plant Mini kit (Qiagen). Reverse transcription was performed using MMLV RT kit (Evrogen) with random decanucleotide primers followed by RT-PCR (primers are listed in supplementary table S4, Supplementary Material online). Annotation of a complete sequence (for *Petrosavia*) and contigs (for *Japonolirion*) was performed using DOGMA (Wyman et al. 2004) with further manual checking and correction. For visualization of gene content and order web-based tool, GenomeVx was used (http://wolfe.ucd.ie/GenomeVx/, last accessed January 13, 2014). For repeat content and synteny analysis, plastome sequences were truncated to retain only one IR copy and used in all kinds of analysis in this form. To determine the exact position of IR copies in sequence, we have performed Blast alignment of each plastome to itself. A Blast match was considered to describe IR if its length was more than 500 bp with identity more than 95%, and two sequences constituting the match were reverse complement to each other. The IR copy situated at the end of plastome sequence is removed. To detect repeats, Vmatch 2.2.1 (http://www.vmatch.de/, last accessed January 13, 2014) was used. We searched for repeats longer or equal to 30 bp with similarity no less than 90% and no more than 10 differences (which can arise from mismatches, insertions, and deletions) totally. Both direct and inverted repeats were searched for, without limitations for maximal distance between two repeat instances and not allowing two repeat instances to overlap (-l 30 1 -identity 90 -e 10 -seedlength 10 -d -p). The estimation of syntenic blocks was performed with mauveAligner from Mauve 2.3.1 (Darling et al. 2010). Minimal weight of colinear block to be considered was taken 300 and seed size was nine nucleotides. Inversions of whole single copy region were not treated as rearrangements because it was demonstrated that chloroplast DNA exists in two forms relative to the orientation of SSC versus LSC (Palmer 1983; Martin et al. 2013). In some cases (for small blocks and/or regions with low sequence conservation), the estimates of syntenic blocks number can be inaccurate. To optimize the alignment, the reference plastome was chosen for each evolutionary lineage (*Amborella trichopoda* for basal angiosperms, *Liriodendron tulipifera* for magnoliids, *Nandina domestica* for basal eudicots, *Arabidopsis thaliana* for rosids, *Nicotiana tabacum* for asterids, and *Acorus calamus* for monocots), and all sequences from representatives of this group were aligned against it. All reference plastomes are completely colinear one to another (with the exception of minor shifts of the IR-SC border). Phylogenetic analysis was performed using a set of sequences of 37 protein coding genes from 93 angiosperm plastid genomes. We considered only plastid genes present in *Petrosavia* and in other plants. Nucleotide sequences were aligned according to corresponding aminoacid alignment produced by MUSCLE (Edgar 2004), and frameshift mutations were corrected manually. The most variable and gap-rich positions were excluded from the alignment using the GBLOCKS program (Castresana 2000). We used the “softest” settings and reduced 52,638 positions of nucleotide alignment to 30,423. Phylogenetic trees were reconstructed using maximum likelihood approach as implemented in RAxML (Stamatakis 2006) for both nucleotide and aminoacid alignments. GTR + G model was selected by the Akaike information criterion (AIC) in Modeltest (Posada and Crandall 1998) for nucleotide sequences, and JTT + F + G model was selected by the AIC in ModelGenerator (Keane et al. 2006). ML branch support was assessed via 100 nonparametric bootstrap pseudoreplicates, using the “rapid” bootstrap approach. Comparison of nucleotide substitution relative rates was performed using the GRate program (Müller 2003). A topology of the NJ tree rooted with *A. trichopoda* and a nucleotide substitution model selected in Modeltest were used.

## Supplementary Material

Supplementary figures S1 and S2 and tables S1–S4 are available at *Genome Biology and Evolution* online (http://www.gbe.oxfordjournals.org/).

Supplementary Data
