# Supplementary material for: The Plastid Genome of Mycoheterotrophic Monocot Petrosavia stellaris Exhibits Both Gene Losses and Multiple Rearrangements
Source: Genome Biol Evol. 2014 Jan 6;6(1):238–46. doi: 10.1093/gbe/evu001 (PMC3914687; doi:10.1093/gbe/evu001)
Supplement: Supplementary Data [file supp_6_1_238__index.html]

The plastid genome of mycoheterotrophic monocot Petrosavia stellaris exhibits both gene losses and multiple rearrangements. — The Plastid Genome of Mycoheterotrophic Monocot Petrosavia stellaris Exhibits Both Gene Losses and Multiple Rearrangements — Supplementary Data 

# The Plastid Genome of Mycoheterotrophic Monocot *Petrosavia stellaris* Exhibits Both Gene Losses and Multiple Rearrangements

## Supplementary Data

files

**Files in this Data Supplement:**

- Supplementary Data - jpg file
- Supplementary Data - doc file
- Supplementary Data - xls file
- Supplementary Data - jpg file
- Supplementary Data - doc file
- Supplementary Data - xls file
