# Supplementary figures and images for: The Plastid Genome of Mycoheterotrophic Monocot Petrosavia stellaris Exhibits Both Gene Losses and Multiple Rearrangements
Source: Genome Biol Evol. 2014 Jan 6;6(1):238–46. doi: 10.1093/gbe/evu001 (PMC3914687; doi:10.1093/gbe/evu001)

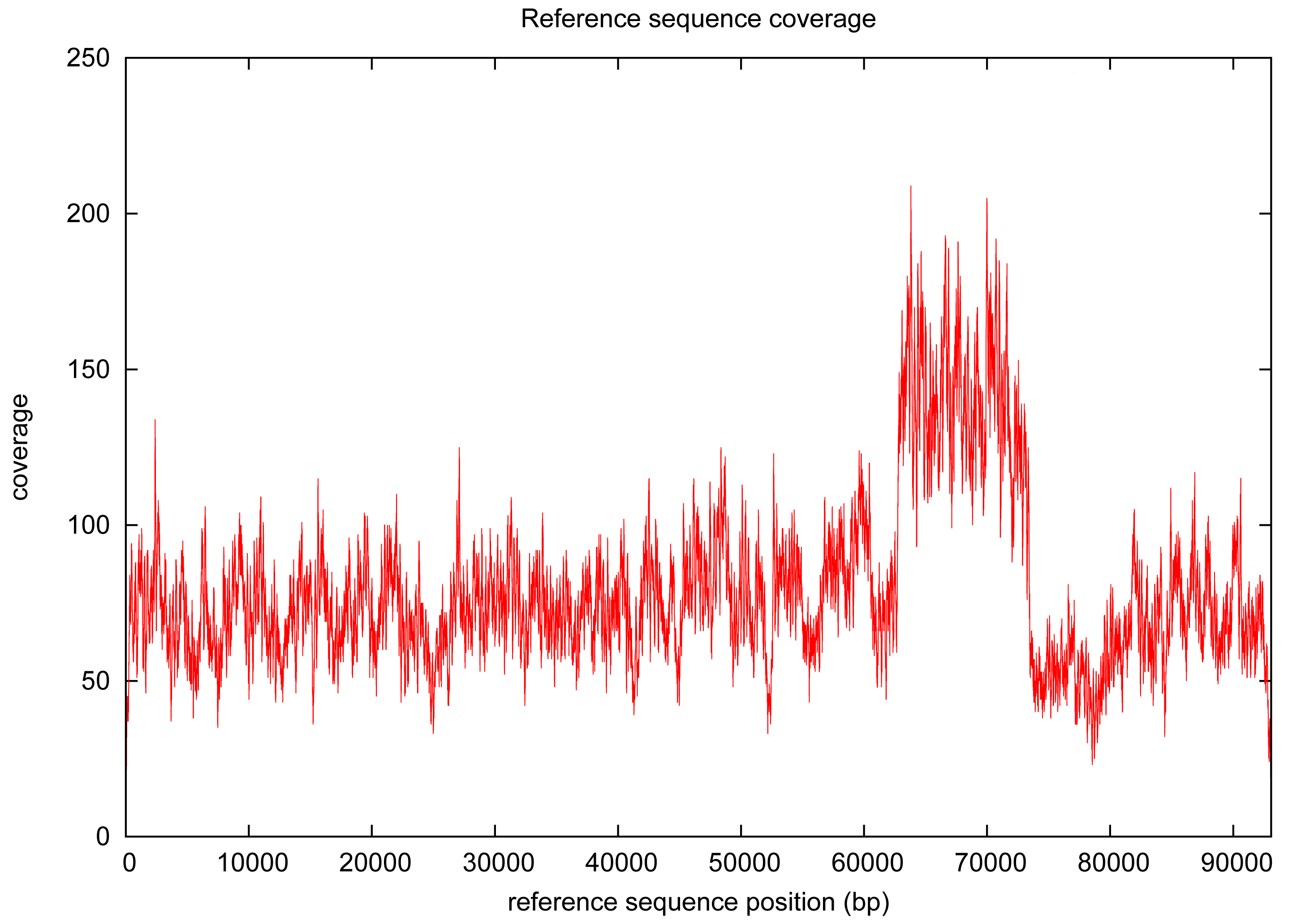

Supplement: Supplementary Data [file supp_evu001_Suppl_fig_1_coverage.jpg]

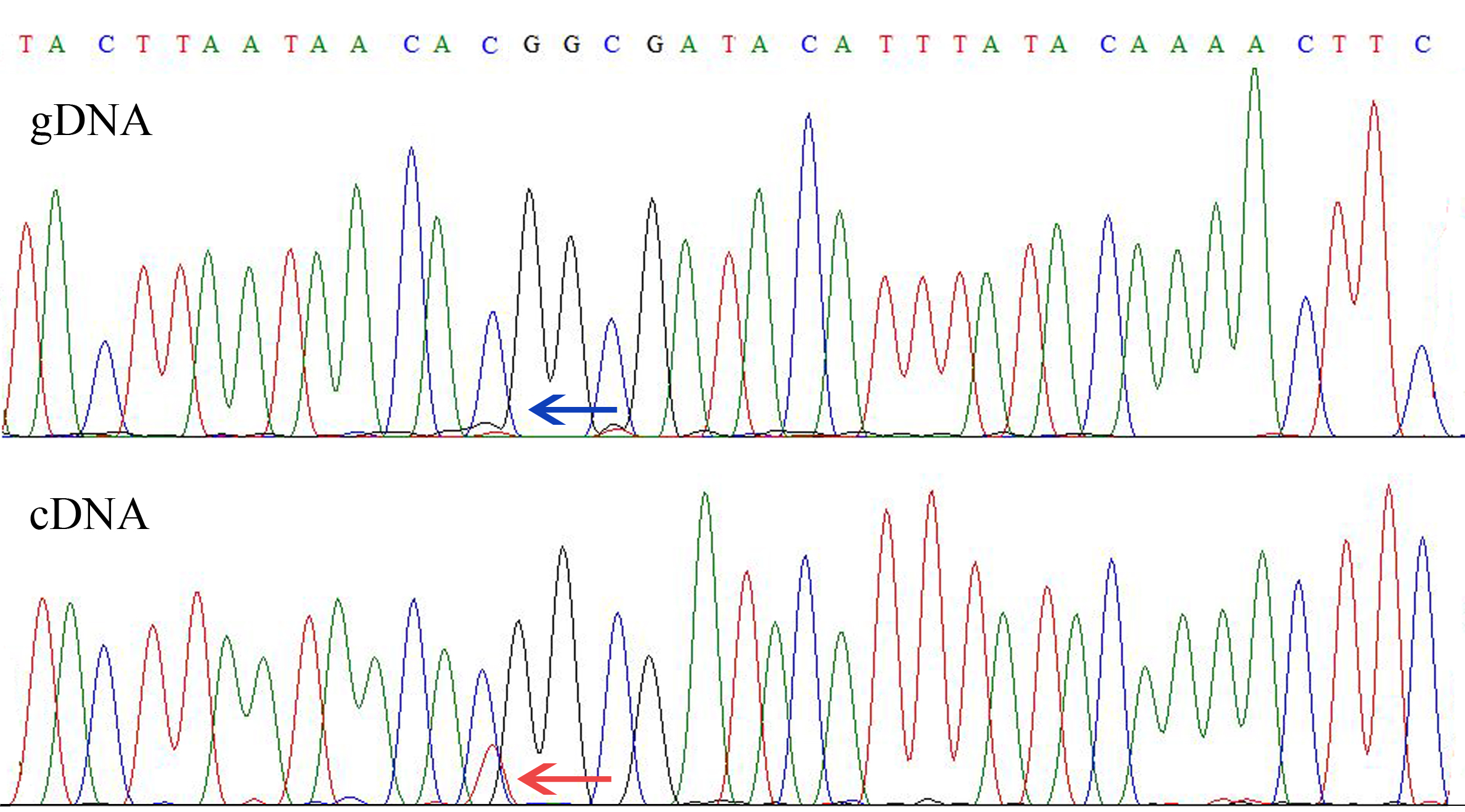

Supplement: Supplementary Data [file supp_evu001_Suppl_Fig2_redact.jpg]
