# Supplementary material for: The Plastid Genome of Mycoheterotrophic Monocot Petrosavia stellaris Exhibits Both Gene Losses and Multiple Rearrangements
Source: Genome Biol Evol. 2014 Jan 6;6(1):238–46. doi: 10.1093/gbe/evu001 (PMC3914687; doi:10.1093/gbe/evu001)
Supplement: Supplementary Data [file supp_evu001_suppl_table_4_primers.doc]

| Primer name | Sequence | Orientation | Position |
| --- | --- | --- | --- |
| *Petrosavia stellaris* | | |  |
| Petro-atpB_C3-rev-F1 | GAATTTCGTATCTAACTGTATAAAGG | + | 56190 -56215 |
| Petro-C34_rrn23-F | GGAAGATCAGCCTGTTATCC | - | 79486 – 79505 |
| Petro-C6_trnI-GAU-R | CTAACAACGCATCTTCACAGAC | + | 75309 – 75330 |
| Petro-ycf2-C3_rev-F | CACGATCGAGACAATTGGCTGA | + | 46349 – 46370 |
| Petro-ycf2-C3_rev-R | CTGTCAAAGTAAGGAACCATGG | - | 47090 – 47111 |
| Petro-atpB_C3-rev-F | CATCTGTAGCACTCATAGCTAC | + | 56378 – 56399 |
| Petro-rbcL_C3-rev-R | GTTCTTTGCGGATAATCCCAAT | - | 57930 – 57951 |
| Petro-C2-atpH-F | GTTACTTCGACCCGATAGATC | + | 19651 – 19675 |
| Petro-C20_atpA-R | CCTGTTTGGAGAGATCATCATA | - | 22561 – 22582 |
| Petro-C20-trnQ-F | GTTACTCGTAGGTTCGAATCCT | + | 26147 – 26168 |
| Petro-C3rev-rps16-trnQ-R | CGAAAGTCCTTTATTACCGAA | - | 26865 – 26885 |
| Petro-C2_trnL-UAA-F | GTTCAAGTCCCTCTATCCCCAAT | + | 2105 – 2127 |
| Petro-C2-trnF-psaB-R | AGAAAAGGGCACATAATCTCATT | - | 2747 – 2769 |
| Petro-rpl20+F | GCAGTAACTCACAAGAATAAGGTAT | -/+ | 63583 – 63607/102954 – 102978 |
| Petro-rps4-R | AGCGACAATTACTTAGATATGTGCAT | - | 392 – 417 |
| Petro-rpl33-F | ATAAAGAAATAGATCGAGCGTAACAT | + | 62132 – 62157 |
| Petro-trnV-GAC-F | AGTTCGAGCCTGATTATCCCTA | +/- | 62909 – 62930/93631 – 93652 |
| Petro-rrn16-R | AGGACTACTGGGGTATCTAAT | - | 73865 – 73885 |
| Petro-trnA-UGC-F | AACTACGAGATCACCCCAAG | + | 76426 – 76445 |
| Petro-rpl32-R | AAGGTTTTCTTACTTGTTTGCC | + | 92879 – 92900 |
| Petro-rrn23-F1 | TTTAACGGTTTAGGCTGCTC | - | 77267 – 77286 |
| Petro-rbcL-F | CTAACATGTTTACTTCCATTGTAGG | + | 57740 – 57764 |
| Petro-rbcL-R1 | TTTTGAATTATGCGCACTTATT | - | 58838 – 58859 |
| Petro-ycf2-R1 | AACGAGATATCCAGCAACAAGA | - | 47431 – 47452 |
| Petro-petD-F | GACTCTTACTCAAAAGGTCCAAT | + | 34827 – 34849 |
| Petro-petD-R | TAGCTAACCTGGCAGAACCAAT | - | 35313 – 35334 |
| Petro-rpl20+F1 | CGAACATCTTTCATTTCAATAATAATAAA | -/+ | 63307 – 63335/103226 – 103254 |
| Petro-trnA-UGC-R | AGAATGCTGGTTTGTTTTAAGAAC | - | 76561 – 76584 |
| Petro-clpP-1exon-F | ATGCCCATTGGTGTTCCAAAAGTA | -/+ | 66885 – 66908/99653 – 99676 |
| Petro-clpP-3exon-R | TACACCGAGTCGACAGTATTTTC | +/- | 64795 – 64817/101744 – 101766 |
| Petro-rps12-F* | CAGCTTATTAGAAACACAAGACAG | -/+ | 64616 – 64639/101922 – 101945 |
| Petro-rps12-R* | CTTGTTGACGATCCTTTACTC | +/- | 70824 – 70844/95717 – 95737 |
| Petro-rpl23-F | GAATGCATTACAGACGTATGATCATT | - | 42823 – 42848 |
| Petro-rpl2-R | CTCTATGCCCTGCGGTAATGATTC | + | 42597 – 42620 |
| *Japonolirion osense* | | | |
| Ja_os_rps19-F | CGAGACCAAGTTACTATTATTTCTT |  |  |
| Ja_os-trnH-R | TCACAATCCACTGCCTTGA |  |  |
| Ja_os_atpB-rbcL-F | GATTGGGTTGCGCCATATATATCAA |  |  |
| Ja_os_rbcL-R | TCAATTTGTAATCTTTAACACCAG |  |  |

Supplementary table 4. Sequences of primers used for contig joining, assembly validation and cDNA sequencing.

* - this pair amplifies cDNA only
