# Supplementary material for: The Plastid Genome of Mycoheterotrophic Monocot Petrosavia stellaris Exhibits Both Gene Losses and Multiple Rearrangements
Source: Genome Biol Evol. 2014 Jan 6;6(1):238–46. doi: 10.1093/gbe/evu001 (PMC3914687; doi:10.1093/gbe/evu001)
Supplement: Supplementary Data [file supp_evu001_suppl_table1_Ja_os_contigs.doc]

| scaffold № | length, bp | genes | GC content, % | accession number |
| --- | --- | --- | --- | --- |
| 6 | 52466 | rps16-atpB | 36.7 | KF454707, KF454714 |
| 1* | 26533 | trnH-ycf1 | 42.9 |  |
| 20 | 23498 | cemA-rps19 | 35.7 | KF454711, KF454712 |
| 7 | 18560 | ycf1-ndhF | 32.2 | KF454708, KF454709 |
| 12 | 3401 | psbA-matK | 36.4 | KF454710 |
| 9 | 2172 | accD | 33.7 | KF454713 |
| 27** | 1875 | rbcL | 41.1 |  |

Supplementary table 1.

*Japonolirion osense* assembly characteristics.

* - joined by PCR with scaffold 20, ** - joined by PCR with scaffold 6.
